# Self-activating air filtration device for aerosol control during luminal instruments drying in the sterile processing department

**DOI:** 10.1371/journal.pone.0331485

**Published:** 2025-09-10

**Authors:** Wei Zheng, Ying He, Ping Gui, Xiaoxue Sun

**Affiliations:** 1 Sterile Processing Department, Sichuan Clinical Research Center for Cancer, Sichuan Cancer Hospital &Institute, Sichuan Cancer Center, Affiliated Cancer Hospital of University of Electronic Science and Technology of China, Chengdu, China; 2 Department of Nursing, Sichuan GEM Flower Hospital, North Sichuan Medical College, Chengdu, China; 3 Sterile Processing Department, Sichuan GEM Flower Hospital, North Sichuan Medical College, Chengdu, China; SKUMS: Shahrekord University of Medical Science, IRAN, ISLAMIC REPUBLIC OF

## Abstract

**Background:**

Luminal instruments are characterized by their slender internal lumens, which make them particularly challenging to clean and dry. A common drying method used by Sterile Processing Department (SPD) technicians involves blowing high-pressure air into one end of the lumen to expel moisture. However, this process generates a significant amount of aerosols that may contain bacteria, viruses, and other microorganisms. These aerosols can increase the microbial load in the SPD environment, leading to secondary contamination of other medical devices and posing health risks to SPD staff. Current methods used in SPD to reduce aerosols, such as gauze pockets and perforated boxes, have shown limited effectiveness.

**Methods:**

This study designed and fabricated a self-activating air filtration device, which primarily consists of four components: a centrifugal fan, a high-efficiency air filter, an acrylic box, and a diffuse reflective photoelectric switch. The total cost of the device is approximately $25. To verify the functionality of the device, we selected 20 sets of endoscopic instrument packs, 40 suction tubes, and 20 plastic hoses. The device’s efficacy in reducing airborne dust particles and biological aerosols was evaluated, and a simple smoke test was conducted to assess its reliability.

**Results:**

Compared to results obtained without a HEPA filter, the self-activating air filtration device achieved a substantial reduction in airborne dust particles across various sizes: 79.34% for 0.3μm, 78.66% for 0.5μm, 76.58% for 1.0μm, 76.15% for 2.5μm, 72.55% for 5.0μm, and 75.00% for 10μm particles, with an average reduction of 76.36% (calculated using the median). No bacterial growth was observed on the ten culture plates sampled using the device, whereas five out of the ten culture plates sampled without the HEPA filter exhibited bacterial growth, resulting in a positivity rate of 50%. The differences were statistically significant. Additionally, the smoke test confirmed that the device effectively prevented contaminated air from escaping through the top opening of the acrylic box.

**Conclusion:**

The self-activating air filtration device significantly reduces the aerosols generated during the drying of luminal instruments, thereby mitigating the risks that aerosols pose to both the environment and staff health. Its automatic on-off functionality and low cost of use and maintenance make it a valuable solution for aerosol control in SPD and other hospital settings.

## Introduction

The Sterile Processing Department (SPD) is responsible for the cleaning, disinfection, and sterilization of reusable medical devices, playing a vital role in controlling hospital infections and ensuring medical safety [1–3]. There are numerous types of medical devices used in hospitals, including vascular forceps, tissue forceps, scissors, power tools, and luminal instruments. Puncture needles, suction tubes, and endoscopic surgical instruments all fall under the category of luminal instruments, which have slender internal lumens [4,5]. This unique structure increases the difficulty of cleaning, disinfection, and drying [6–9]. The SPD can clean and disinfect luminal instruments using either manual or mechanical methods [10–12]. Subsequently, it is essential to dry the luminal instruments, especially their internal lumens, as residual moisture inside can not only promote bacterial growth but also lead to wet packs during steam sterilization or interrupt hydrogen peroxide gas sterilization processes [10,13–16]. Automated washer-disinfectors, hot air drying cabinets, and vacuum drying cabinets can all be used for drying luminal instruments. Additionally, high-pressure air guns using compressed medical-grade air is a common drying method [10,11,17]. By blowing high-pressure air into one end of the lumen, the moisture inside the lumen is rapidly expelled, thus achieving drying. During the medical device inspection and packaging process, SPD technicians use high-pressure air guns to blow through the internal channels of each luminal instrument. This serves two purposes: both to facilitate drying and to check whether the interior of the luminal instruments is thoroughly cleaned (any blood, foreign objects, or residual human tissue will be blown out by the high-pressure air gun) [11,18,19].

However, the use of high-pressure air guns to dry luminal instruments can generate aerosols. When high-pressure air is blown into one end of the luminal instrument, the airflow rapidly travels through the internal channel of the instrument and exits from the other end, breaking the residual moisture inside into tiny droplets and forming aerosols, which then disperse into the SPD environment [20–23]. Since disinfection cannot kill all bacteria, the interior of luminal instruments after cleaning and disinfection may not only have residual moisture but also residual bacteria. Moreover, if the cleaning or disinfection process is not strictly followed by the SPD technicians, the luminal instruments may also retain viruses, stains, and blood, all of which can become part of the aerosols [22–26]. The generation of aerosols poses serious contamination and health threats. On one hand, microorganisms such as viruses and bacteria can spread through the air, increasing the microbial load in the environment of the SPD and potentially causing secondary contamination of other cleaned and disinfected medical devices. On the other hand, for SPD technicians, inhaling aerosols containing microorganisms may cause respiratory infections, allergies, and other health problems, posing a direct threat to their physical health [22,23,27–29].

The physical and chemical properties of aerosols, along with their associated health and environmental risks, as well as their generation, diffusion, detection, and control measures, have all garnered substantial attention and research efforts. In hospital settings, numerous studies have explored the distribution of aerosols and control measures in wards, elevators, waiting areas, dental clinics, operating rooms, ICUs, and ambulances [30–34]. Researchers have proposed various methods to minimize the occurrence or spread of aerosols, such as using plexiglass barriers, the combination of curtains and purifiers, portable air filtration, the intubating box, and aerosol barrier devices, all of which have shown promising results [35–39]. In the SPD, some technicians make gauze pockets, placing the outlet end of luminal instruments into the gauze pocket during high-pressure air gun drying, using the adsorption and filtration properties of gauze to capture droplets and microorganisms in aerosols [40]. Additionally, SPD technicians have also fabricated cubic boxes with openings on the top or side for inserting luminal instruments; medical absorbent paper or gauze is placed inside the box. During the drying of luminal instruments, the outlet end of the instrument is inserted into the box, and the absorbent paper and gauze are expected to reduce aerosol contamination [41]. Both gauze pockets and self-made boxes rely on absorbent paper and gauze for aerosol adsorption; however, the pore size of absorbent paper and gauze is large, indicating a limited capacity to adsorb aerosols. More importantly, the internal volume of these gauze pockets and boxes is very small. When a high-pressure air gun is used to dry luminal instruments, the internal space is filled almost instantaneously, after which the air laden with a large volume of aerosols disperses directly into the room, contaminating the indoor environment. The primary purpose of SPD technicians using gauze pockets and perforated boxes when drying luminal instruments with a high-pressure air gun is to observe whether obvious blood or foreign residues are present inside the luminal instruments, because these contaminants will be clearly adsorbed onto the gauze and thus be detected by SPD technicians. A more effective method for reducing aerosols is the use of large aerosol-control cabinets, the structure of which is similar to that of a range hood, with an extraction fan and a high-efficiency air filter as the core components [42]. However, these cabinets are expensive, bulky, and have stringent installation requirements, making their installation and use extremely inconvenient in SPD, especially in the inspection and packaging area.

Despite significant research achievements in aerosol control both domestically and internationally, deficiencies persist in studies targeting aerosol containment during the drying of luminal instruments within the SPD. The methods currently adopted to mitigate aerosol hazards remain insufficiently effective. This study therefore aims to address the shortcomings of existing approaches by designing and fabricating a highly efficient, reliable, and cost-effective self-activating air filtration device. This device is intended to substantially reduce aerosol generation during the drying of luminal instruments, thereby lowering the risk of healthcare-associated infections, safeguarding patient safety, and fostering a safe and healthy working environment for SPD personnel.

## Materials and methods

### Device design and fabrication

**Fig 1:**
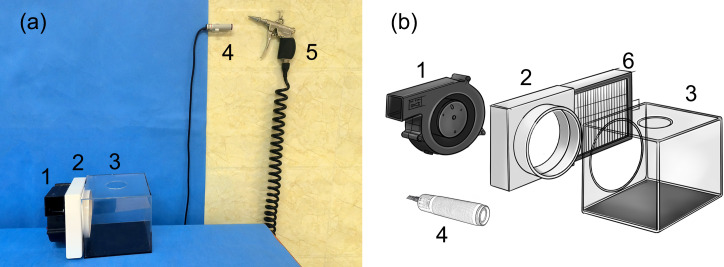
The actual photo and the schematic diagram of the self-activating air filtration device. (a) The actual photo and (b) the schematic diagram. 1. Centrifugal fan; 2. Connector; 3. Acrylic box; 4. Diffuse reflective photoelectric switch; 5. High-pressure air gun; 6. HEPA filter.

The self-activating air filtration device mainly consists of four parts: a centrifugal fan, an air filter, an acrylic box, and an automatic on-off induction component. These components work in concert to effectively control aerosols. The schematic diagram and the actual photo of the device are shown in Fig 1.

*Centrifugal fan:* The centrifugal fan measures 14 cm in length and width, with a thickness of 4 cm. It features an air intake channel and an exhaust port structure. The intake channel has a diameter of 8 cm, and the exhaust port stands 4.7 cm in height. The fan operates at a voltage of 12 V, with a current of 1.5 A, and a rotational speed of 3,000 revolutions per minute. It delivers an airflow of 1,550 liters per minute and generates noise at 63 dBA (measured at a distance of 1 meter). The working principle of the fan involves the motor driving the blades to rotate at high speed. Air is drawn into the intake channel and, under the influence of centrifugal force, is flung to the outer edge of the blades and expelled through the exhaust port.

*Air filter:* The air filter comprises a high-efficiency particulate air (HEPA) filtration unit with a filtration rating of H12 and its associated connector. The HEPA filtration unit measures 14.5 cm in length and width, with a thickness of 2.5 cm. Its filtration mechanism relies on the combined effects of several phenomena, including interception, inertial impaction, diffusion, gravitational settling, and electrostatic attraction. The mounting hardware is made of plastic, measuring 15 cm in length and width, and 3.5 cm in thickness. One side is fully perforated to facilitate the installation and removal of the HEPA filtration unit. The base features a circular opening with a diameter of 11 cm at the center, which connects to the air intake channel of the centrifugal fan. The top surface includes a protruding cylindrical structure with an inner diameter of 11 cm (and an outer diameter of 11.5 cm).

*Acrylic box:* The acrylic box measures 15 cm in length, width, and height. The top and sides are made of transparent acrylic material to facilitate the observation of internal operations, while the base is composed of black opaque acrylic. A 5-cm-diameter hole is located at the center of the box’s top for inserting instruments during high-pressure air gun drying of luminal instruments. Additionally, an 11.5-cm-diameter hole is present on one side for connecting the HEPA filter.

*Automatic On-Off Induction Component:* We selected a diffuse reflective photoelectric switch (E3F-DS30P1-PNP, normally closed type) as the induction module, which integrates an emitter and a receiver and has an effective sensing distance of 7–30 cm. The emitter continuously emits infrared light, which is reflected back and detected by the receiver when it encounters an object. In the absence of an object, the receiver detects no reflected light. Mounted on the wall approximately 10 cm beside the high-pressure air gun, the switch controls automatic start-up and shut-down of the entire device by sensing the presence or absence of the gun. When the air gun remains in place, it blocks the switch, the circuit remains open, and the device stays off. When the gun is removed, the blockage is eliminated, the receiver no longer detects reflected light, the circuit closes, and a signal is sent to activate the device. Once the air gun is returned to its original position, the receiver again detects reflected light, the circuit opens, and the device ceases operation.

**Fig 2:**
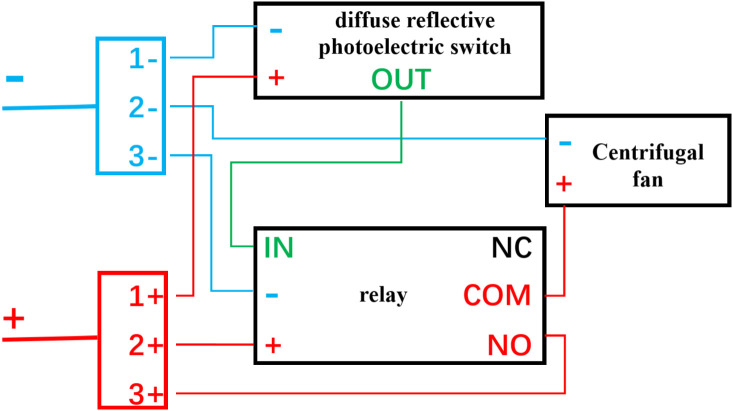
The wiring diagram of the self-activating air filtration device. Positive pole (red), negative pole (blue), and signal wire (green).

The side opening of the acrylic box connects to the cylindrical structure of the air filter. The centrifugal fan is attached to the other side of the air filter connector with glue. The power supply, centrifugal fan, and diffuse reflective photoelectric switch are connected through a relay. The relay, responsible for signal reception and circuit connection, features a delay function that enables immediate activation upon receiving a start signal and a 15-second delay before deactivation upon receiving a stop signal(The duration of the delay can be set anywhere between 0 and 30 minutes.). The wiring diagram of the self-activating air filtration device is shown in Fig 2.

### Device application

When the technician retrieves the high-pressure air gun from its wall mount to initiate the drying procedure of luminal instruments, this action instantaneously activates the diffuse reflective photoelectric switch affixed to the side of the air gun, which then transmits a signal. Upon receipt of this signal, the relay facilitates circuit closure, thereby activating the centrifugal fan. The centrifugal fan continuously draws air from within the acrylic box through the HEPA filter and expels it into the ambient environment, establishing a negative pressure within the acrylic box. The technician positions the high-pressure air gun at one end of the luminal instrument, with the opposite end of the instrument inserted into the aperture on the top of the acrylic box. As the high-pressure air gun injects air into the luminal instrument, the resultant aerosols—comprising moisture, bacteria, and particulate matter—are filtered through the HEPA filter prior to being discharged. Upon completion of the drying process, the technician returns the high-pressure air gun to its designated position. The induction switch detects the restoration of the air gun to its original position and subsequently signals the relay to maintain circuit continuity for an additional 15 seconds before deactivating the centrifugal fan. The device then enters a standby mode, ready for subsequent use.

### Device verification

Given the limited effectiveness of existing SPD strategies for reducing aerosols generated during the drying of luminal instruments—namely gauze pockets and perforated boxes—and the poor operability of large aerosol-control cabinets, together with the complexity of aerosol sampling across different methods, we did not compare the self-activating air filtration device with current practices during efficacy validation; instead, we evaluated only its own capacity to reduce aerosols.

To validate the functionality of the self-activating air filtration device, a diverse range of luminal instruments were selected, encompassing 20 sets of endoscopic instrument packs from gynecology, thoracic surgery, general surgery, and urology departments (each set comprising 10–20 luminal instruments, with 10 sets undergoing manual cleaning and 10 sets undergoing mechanical cleaning), 40 suction tubes of varying lengths, diameters, and shapes, and 20 plastic hoses (with an inner diameter of 1 cm and a length of 180 cm). Testing commenced within 30 minutes after cleaning and disinfection of these instruments. The experiments were conducted in the packaging area of the SPD, where the ambient temperature was maintained between 20°C and 24°C, and the relative humidity was controlled within the range of 40% to 50%.

The efficacy of the self-activating air filtration device was assessed using the number of airborne dust particles and bacteria as evaluation metrics. Airborne dust particles were quantified using an MKS 800 particle counter, which operates at a flow rate of 1 L/min and performs continuous measurements for 2 minutes per session. This device is capable of simultaneously measuring the concentration of airborne dust particles in six size fractions: 0.3μm, 0.5μm, 1.0μm, 2.5μm, 5.0μm, and 10μm. Bacterial counts were determined using the settle plate method, employing ordinary nutrient agar plates exposed for 5 minutes. Following exposure, the plates were incubated at 36°C for 48 hours, followed by colony enumeration [43].

**Fig 3:**
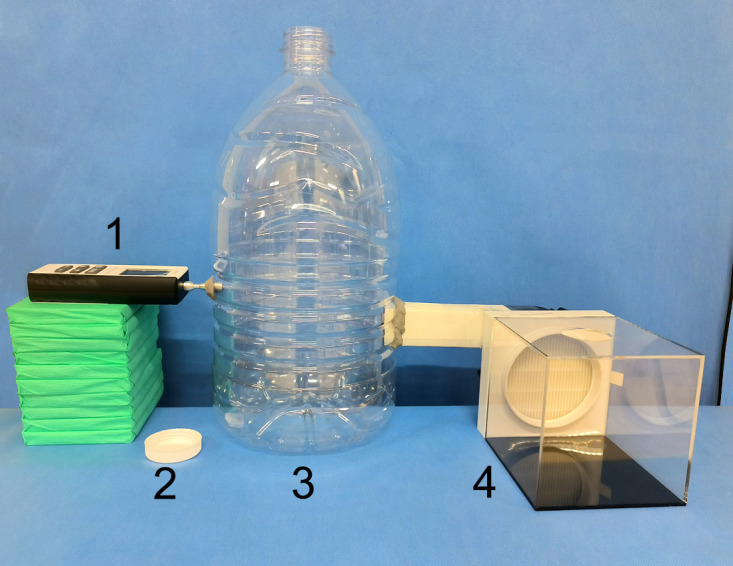
Simplified sampling apparatus for the validation of the self-activating air filtration device. The air particle counter (1) and the exhaust port of the centrifugal fan (4) are connected to the side of the bucket (3) and sealed. The lid (2) is opened during testing and closed during sampling.

To facilitate the sampling process for the validation procedure, a simplified sampling apparatus was developed. This apparatus primarily consists of a transparent plastic bucket with an approximate volume of 13 L and a removable lid. The exhaust port of the centrifugal fan and the probe of the particle counter are connected to the side of the bucket, respectively (Fig 3). The lid is removed from the bucket, and a single culture plate (without a lid) is placed at the bottom of the bucket. A technician randomly selects one set of endoscopic instrument packs, two suction tubes, and one plastic hose to perform the drying operation of luminal instruments using a high-pressure air gun, with the blowing time ranging from 90 to 120 seconds. After the operation is completed, the high-pressure air gun is returned to its original position. Once the centrifugal fan has ceased operation, the lid of the plastic bucket is immediately replaced. The particle counter is then activated to conduct the test and record the results. Five minutes after the lid is replaced, the culture plate is removed, covered with its lid, and labeled. This procedure is repeated 10 times. Subsequently, the HEPA filter is removed from the self-activating air filtration device, and the openings on the connector, which result from the removal of the HEPA filter, are sealed with transparent tape. The test was repeated 10 times following the aforementioned method. Finally, the 20 culture plates are placed into the incubator for cultivation.

To further ascertain whether the self-activating air filtration device fully exhausted the air within the acrylic box, thereby preventing the escape of aerosol-laden air through the top opening of the box, we conducted additional validation tests. Given the complexity of accurately calculating the pressure conditions and airflow direction at the top opening of the acrylic box—owing to the interplay of multiple factors such as the shape of the box, the pressure of the compressed air, the internal diameter of the high-pressure air gun, and the performance characteristics of the HEPA filter and centrifugal fan—we employed a simple smoke test method to visually assess the airflow direction at the top opening. The centrifugal fan was activated, and the nozzle of the high-pressure air gun (set at a pressure of 0.8 MPa) was inserted into the top opening of the acrylic box to blow air. A smoke tablet (with a diameter of 2 cm, thickness of 0.5 cm, and composed of 70% ammonium chloride, 15% rosin, and 15% flour, which produces a large amount of white smoke when ignited) was placed and ignited 2 cm outside the edge of the opening. The trajectory of the white smoke was meticulously observed, and this test was repeated five times to ensure the consistency and reliability of the results.

### Statistical analysis

Data were analyzed using R 4.5.1. Categorical variables were compared between groups with Fisher’s exact test. Continuous data that were not normally distributed are presented as M (P_25_, P_75_), and inter-group comparisons were performed using the Mann–Whitney U test. A p-value < 0.05 was considered statistically significant.

## Result

### Comparison of airborne dust particle counts

Compared with results obtained without a HEPA filter, the self – activating air filtration device reduced the number of airborne dust particles with sizes of 0.3μm, 0.5μm, 1.0μm, 2.5μm, 5.0μm, and 10μm by 79.34%, 78.66%, 76.58%, 76.15%, 72.55%, and 75.00%, respectively, with an average reduction of 76.36% (calculated using the median). All differences were statistically significant (P ≤ 0.05). See Table 1.

**Table 1:** Comparison of airborne dust particle counts of different diameters before and after using the HEPA filter. (M (P_25_, P_75_)).

Groups	0.3μm	0.5μm	1μm	2.5μm	5μm	10μm
Without a HEPA filter	218199(210064, 225718)	19299(19070, 19697)	1392(1327, 1412)	130(123, 131)	51(48,54)	4(4, 5)
With a HEPA filter	45301(44499, 48234)	4119(4039, 4176)	326(299, 337)	31(28, 32)	14(11, 15)	1(0, 1)
Z value	−3.742	−3.742	−3.742	−3.750	−3.760	−3.875
P value	<0.001	<0.001	<0.001	<0.001	<0.001	<0.001

### Comparison of bacterial counts

In the ten culture plates sampled using the self-activating air filtration device, no bacterial growth was observed. In contrast, among the ten culture plates sampled without using a HEPA filter, five plates exhibited bacterial growth, with a positivity rate of 50%, resulting in the formation of 1, 3, 1, 1, and 2 colony-forming units, respectively. fisher’s exact test yielded p = 0.033 and the difference was statistically significant.

### Smoke test results

**Fig 4:**
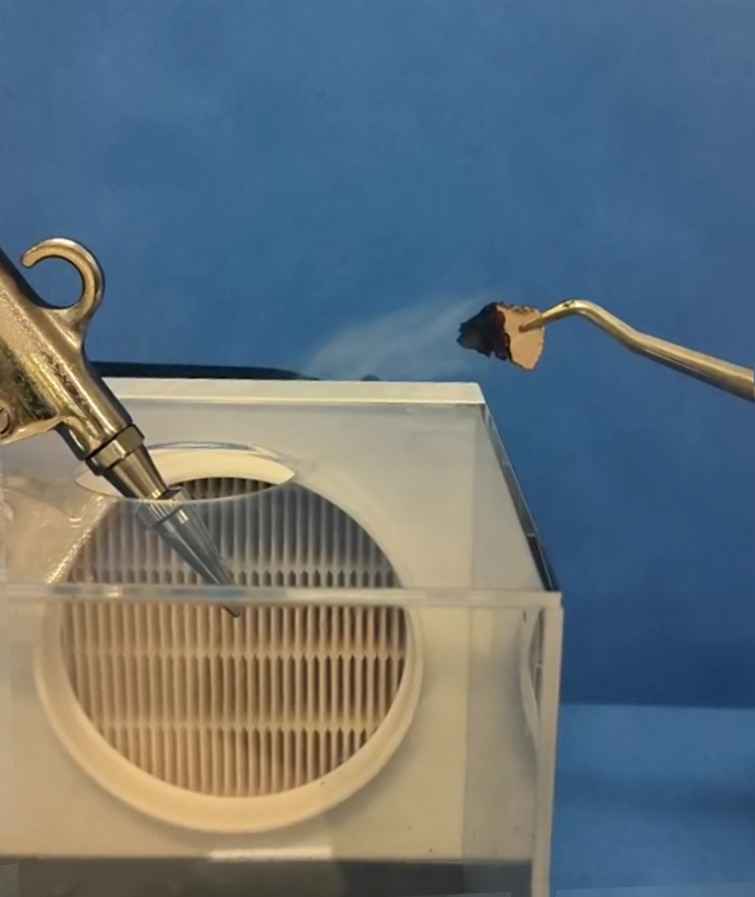
Smoke test. The white smoke being drawn into the acrylic box indicates that the contaminated air within the acrylic box does not escape into the environment.

In the five smoke tests conducted, the white smoke was visibly drawn into the interior of the acrylic box (Fig 4).

## Discussion

The self-activating air filtration device significantly diminishes the aerosols generated during the drying of luminal instruments. The H12-grade HEPA filter within the device is instrumental in reducing aerosol concentrations, with a theoretical filtration efficiency of 99.5% for 0.3-micron particles [44]. However, in our tests, the H12 HEPA filter demonstrated an average filtration efficiency of 76.36% across various particle sizes, which is notably lower than the theoretical value. This discrepancy primarily stems from the difference in face velocity. During performance testing of the H12 HEPA filter, the face velocity was 1.75 cm/s [45]. In contrast, in our experimental setup, the face velocity reached 17.23 cm/s. (Given that the HEPA filter increases the working resistance of the centrifugal fan, the working airflow of the centrifugal fan is calculated at 80% of the maximum value. The filter surface area is 0.12 m^2^, face velocity = airflow/ filter membrane area). The increased face velocity has three consequences: it reduces the dwell time of airborne dust particles of various sizes within the HEPA filter, it enhances the force driving particles through the filter, and it increases the pressure drop across the filter leading to structural changes in the filter membrane [46,47]. These factors collectively contribute to the reduced filtration efficiency observed in our study. Despite this reduction, the 76.36% filtration efficiency still represents a substantial decrease in aerosol and bacterial counts. Specifically, no bacterial colonies were detected on the ten culture plates sampled using the air filtration device. In stark contrast, five out of the ten culture plates sampled without the HEPA filter exhibited bacterial growth, yielding a positivity rate of 50%. The current makeshift solutions employed by SPD technicians, such as gauze pockets and cubic boxes, which lack a reliable filtration mechanism, allow a significant amount of aerosols to disperse into the air. These solutions did not significantly reduce indoor aerosol pollution [40,41]. In contrast, our self-activating air filtration device exhibited a pronounced capacity to reduce aerosol levels, thereby markedly diminishing the associated risks to both the environment and the health of the staff.

The automatic on-off functionality is one of the key advantages of this device. By incorporating a diffuse reflective photoelectric switch on the side of the high-pressure air gun, the device can achieve automatic start and stop. When the high-pressure air gun is removed, the switch detects the change in position and automatically activates the centrifugal fan. Conversely, when the air gun is returned to its original position, the switch senses this change and, after a 15-second delay, automatically deactivates the centrifugal fan. This 15-second delay is specifically designed to more effectively exhaust the contaminated air from the acrylic box and can be adjusted within a range of 0–30 minutes. This automated technology enables seamless operation, enhancing the device’s user-friendliness without significantly increasing the operational steps or workload for SPD technicians. It also ensures that the device is promptly turned off when not in use, conserving energy and reducing noise pollution.

The self-activating air filtration device is distinguished by its straightforward and pragmatic design, which ensures ease of operation and maintenance. The device is composed of four primary components: a centrifugal fan, a HEPA filter, an acrylic box, and a diffuse reflective photoelectric switch. The connections between these components and their operational principles are clear and concise. With the primary design objective of preventing aerosol leakage, the centrifugal fan operates at a high and fixed airflow rate, ensuring complete evacuation of air from the acrylic box even when the high-pressure air gun is set to its maximum working pressure of 0.8 MPa. This capability effectively prevents contaminated air from escaping through the top opening of the acrylic box and ensures that the indoor environment remains free from pollution. The connection between the acrylic box and the air filter connector is designed for repeated disassembly and reassembly, facilitating regular cleaning by SPD technicians. The transparent sides of the acrylic box allow for clear visibility of the interior, enabling technicians to quickly identify any dropped items, stains, or blood crusts, aligning with the functionality described in other studies [23,41]. The diffuse reflective photoelectric switch and the centrifugal fan are connected to the power supply via a relay, with simple and efficient circuit wiring. The HEPA filter is designed for easy installation and removal. The photoelectric switch operates based on the emission and reception of infrared light, providing robust anti-interference performance. It is not triggered by variations in indoor lighting, flying insects, or airborne dust particles. The self-activating air filtration device costs approximately US $25 in total: centrifugal fan $9, HEPA filter $5, acrylic box $5, connector $2, diffuse reflective photoelectric switch $1.5, power adapter $1.5, and delay relay $1. The HEPA filter is the only consumable and must be replaced periodically. While the device is more expensive than gauze pockets and homemade boxes, it is less costly than large aerosol-control cabinets, offering the best cost-effectiveness among the three options.

We recognize that the current experiments are fundamental and relatively straightforward, and thus we have not yet conducted a long-term, comprehensive evaluation of the device’s performance within the SPD. However, as this work serves as an early-stage proof-of-concept, the primary objective was to demonstrate the feasibility of this innovative approach and validate our design through initial testing. Future work will focus on optimizing the shape of the acrylic box, establishing an appropriate replacement schedule for the HEPA filter, and evaluating the performance of higher-grade HEPA filters. Currently, the self-activating air filtration device is positioned on the operating table in the workroom. The acrylic box, which has a height of 15 cm and an opening at the top, necessitates that SPD technicians elevate their hands when using the device, potentially leading to discomfort and fatigue. To address this issue, we plan to redesign the side of the acrylic box closest to the technician as a sloped surface, with the opening relocated to this slope. This modification is anticipated to significantly enhance user comfort. Additionally, given that the HEPA filter may become damp, experience increased resistance, accumulate microbial growth, or sustain damage during use, determining an optimal replacement schedule will be a priority in subsequent research.

## Conclusion

The self-activating air filtration device significantly reduces the generation of aerosols during the drying of luminal instruments, effectively mitigating the risks that aerosols pose to the environment and staff. Additionally, with its automatic on-off functionality, low cost of use and maintenance, the device offers valuable insights and references for aerosol control in sterile processing departments and other hospital settings.

## Supporting information

S1 Data Research Data. (XLSX)
